# Supplementary material for: Characterization of Anthocyanins Including Acetylated Glycosides from Highbush Blueberry (Vaccinium corymbosum L.) Cultivated in Korea Based on UPLC-DAD-QToF/MS and UPLC-Qtrap-MS/MS
Source: Foods. 2025 Jan 9;14(2):188. doi: 10.3390/foods14020188 (PMC11765443; doi:10.3390/foods14020188)
Supplement: Supplementary file 1 [file foods-14-00188-s001.zip › foods-3375018-supplementary.pdf]

Characterization of Anthocyanins Including Acetylated Glycosides from Highbush Blueberry (*Vaccinium corymbosum* L.) Cultivated in Korea Based on UPLC-DAD-QToF/MS and UPLC-Qtrap-MS/MS

Authors: Ju Hyung Kim[1,2], Ryeong Ha Kwon[1], So Ah Kim[1], Hyemin Na[1], Jeong-Yong Cho[2,*] and Heon-Woong Kim[1,*]

Figure S1. Fragmentation (*m/z*, $[M]^+$) patterns of anthocyanins identified in highbush blueberry (**a**, peak **2**) delphinidin 3-*O*-glucoside, (**b**, peak **3**) cyanidin 3-*O*-galactoside, (**c**, peak **4**) delphinidin 3-*O*-arabinoside, (**d**, peak **5**) cyanidin 3-*O*-glucoside, (**e**, peak **6**) petunidin 3-*O*-galactoside, (**f**, peak **7**) cyanidin 3-*O*-arabinoside, (**g**, peak **8**) petunidin 3-*O*-glucoside, (**h**, peak **9**) peonidin 3-*O*-galactoside, (**i,** peak **10**) petunidin 3-*O*-arabinoside, (**j,** peak **11**) peonidin 3-*O*-glucoside, (**k,** peak **13**) peonidin 3-*O*-arabinoside, (**l,** peak **14**) malvidin 3-*O*-glucoside, (**m,** peak **15**) malvidin 3-*O*-arabinoside.

Figure S2. Multivariate analyses in 9 highbush blueberry cultivars based on MS data.

(a) PCA loading plot and (b) OPLS-DA loading plot.

Figure S3. Multivariate analyses in 9 highbush blueberry cultivars based on MS data.

(a) PCA loading plot and (b) OPLS-DA loading plot.

Table S1. VIP values and *p*-values of markers identified between early and mid-seasons by OPLS-DA and t-test

Table S2. Biological activities of various anthocyanins

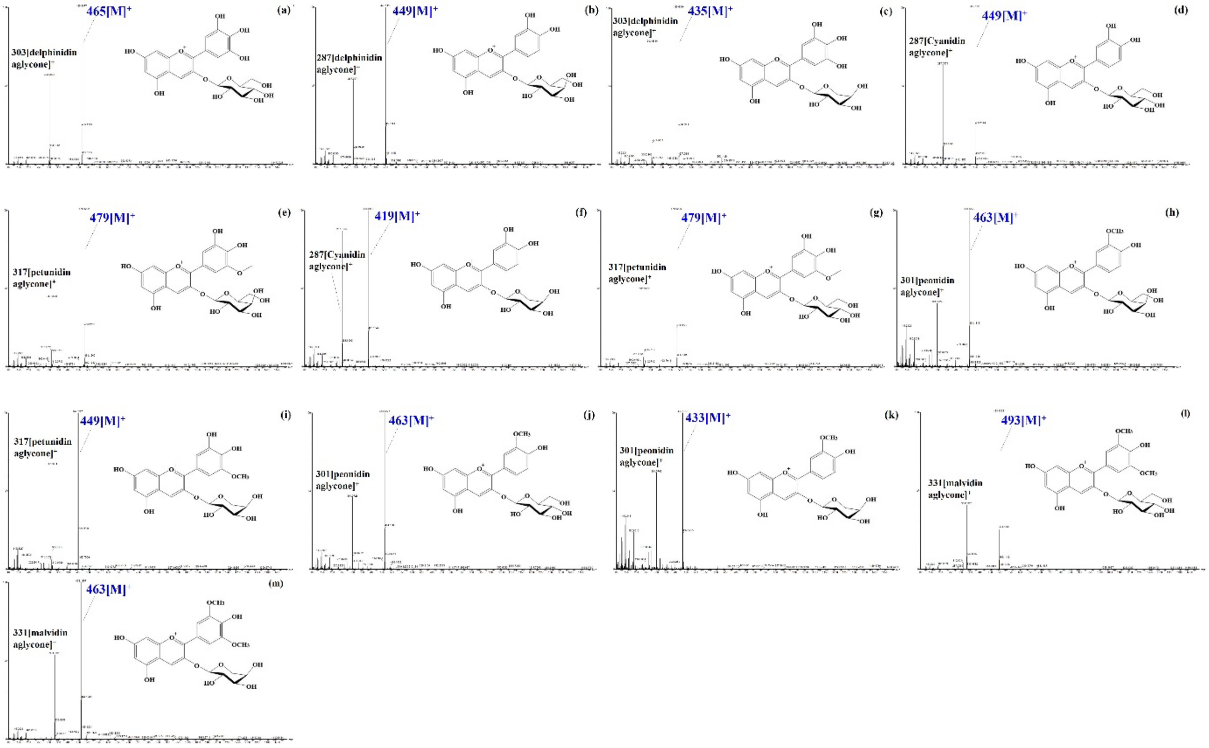

Figure S1. Fragmentation (*m*/*z*, $[M]^+$) patterns of anthocyanins identified in highbush blueberry (**a**, peak **2**) delphinidin 3-*O*-glucoside, (**b**, peak **3**) cyanidin 3-*O*-galactoside, (**c**, peak **4**) delphinidin 3-*O*-arabinoside, (**d**, peak **5**) cyanidin 3-*O*-glucoside, (**e**, peak **6**) petunidin 3-*O*-galactoside, (**f**, peak **7**) cyanidin 3-*O*-arabinoside, (**g**, peak **8**) petunidin 3-*O*-glucoside, (**h**, peak **9**) peonidin 3-*O*-galactoside, (**i,** peak **10**) petunidin 3-*O*-arabinoside, (**j,** peak **11**) peonidin 3-*O*-glucoside, (**k,** peak **13**) peonidin 3-*O*-arabinoside, (**l,** peak **14**) malvidin 3-*O*-glucoside, (**m,** peak **15**) malvidin 3-*O*-arabinoside.

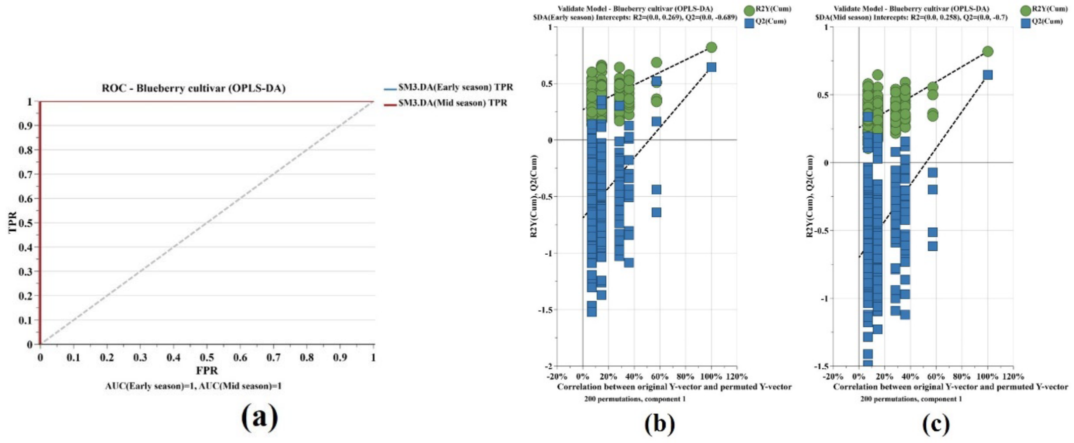

Figure S2. Validation of the OPLS-DA model for highbush blueberry cultivars (a) ROC curve, (b) permutation test for early-season and (c) permutation test for mid-season.

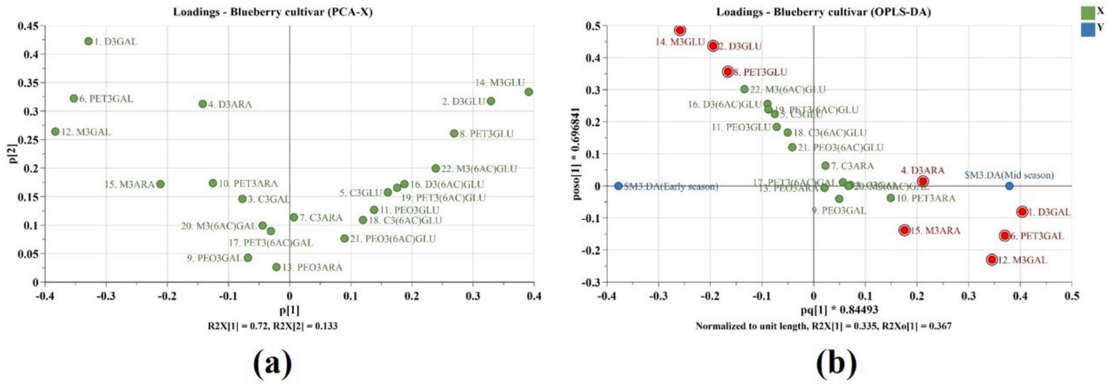

Figure S3. Multivariate analyses in 9 highbush blueberry cultivars based on MS data. (a) PCA loading plot and (b) OPLS-DA loading plot.

Table S1. VIP values and *p*-values of markers identified between early and mid-seasons by OPLS-DA and t-test

Compound	1) VIP-value	2) *p*-value
Delphinidin 3-*O*-galactoside	1.94	1.98E-05
Delphinidin 3-*O*-glucoside	1.84	4.06E-02
Delphinidin 3-*O*-arabinoside	1.79	5.61E-04
Petunidin 3-*O*-galactoside	1.61	5.67E-05
Petunidin 3-*O*-glucoside	1.29	1.86E-03
Malvidin 3-*O*-galactoside	1.08	4.39E-05
Malvidin 3-*O*-glucoside	1.08	6.05E-04
Malvidin 3-*O*- arabinoside	1.02	3.64E-02

1) VIP was obtained from OPLS-DA with a threshold of 1.
2) *p*-value was calculated with t-test

Table S2. Biological activities of various anthocyanins

Anthocyanin.	Model	Does or concentrations	Antioxidant	Mechanism	Reference
Delphinidin 3-*O*-glucoside	HCT-116 colorectal cancer cell line	100-600 μg/mL	Anticancer	induce PD-1 expression inhibition and PD-L1 binding reduction, leading to decreased cancer cell proliferation	[49]
	Molecular Docking, TNF-α Signaling Assay	-	Anti-inflammation	Inhibition TNF-α receptor and TNF-α signaling by direct interaction<br>Docking energy: **-9.21 kcal/mol**	[50]
Cyanidin 3-*O*-glucoside	Co-culture with PBMC and HCT 116 cells	100 μM	Anticancer	inhibition PD-1/PD-L1 expression, reduction VEGF levels and blocking immune evasion and cancer cell survival	[49]
Malvidin 3-*O*-galactoside<br>Malvidin 3-*O*-glucoside	human umbilical vein endothelial (HUVECs)	10 μM	Cardioprotective	Decreased MCP-1, ICAM-1, and VCAM-1 expression<br>Inhibition of IκBα degradation and p65 nuclear translocation	[51]
Cyanidin 3-*O*-rhamnoside	human liver microsomes (pooled)	0-100 μM	Protective hepatocyte	Inhibition CYP3A4 activity (IC_{50} = 44 μM), Regulation CYP2C9, CYP2A6, and CYP2B6	[52]
Delphinidin 3-*O*-rutinoside	human liver microsomes (pooled)	0-100 μM	Protective hepatocyte	Inhibition CYP3A4 activity (down to 35% at 100 µM, IC_{50} = 67 μM)	[52]
Malvidin 3-*O*-(6''-*O*-acetyl)glucoside	β-glucosidase inhibition assay	0-90 μM	Anti-inflammation	inhibition of β-glucosidase activity and enhanced structural changes in the enzyme	[14]
Peonidin 3-*O*-glucoside	Molecular Docking, TNF-α Signaling assay	-	Anti-inflammation	Inhibition the interaction between TNF-α receptor and TNF-α protein, stabilizing the receptor. (Docking energy: **-9.21 kcal/mol)**	[50]
